# Characterization of the Effect of *N*-(2-Methoxyphenyl)-1-methyl-1*H*-benzimidazol-2-amine, Compound 8, against *Leishmania mexicana* and Its In Vivo Leishmanicidal Activity

**DOI:** 10.3390/ijms25010659

**Published:** 2024-01-04

**Authors:** Rocío Nieto-Meneses, Rafael Castillo, Alicia Hernández-Campos, Benjamín Nogueda-Torres, Edgar Oliver López-Villegas, Adriana Moreno-Rodríguez, Félix Matadamas-Martínez, Lilián Yépez-Mulia

**Affiliations:** 1 Departamento de Parasitología, ENCB-Instituto Politécnico Nacional, Mexico City 11340, Mexico; qbprocionieto@gmail.com (R.N.-M.); bnogueda@yahoo.com (B.N.-T.); 2 Unidad de Investigación Médica en Enfermedades Infecciosas y Parasitarias-UMAE Hospital de Pediatría, Centro Médico Nacional Siglo XXI, Instituto Mexicano del Seguro Social, Mexico City 06720, Mexico; 3 Departamento de Farmacia, Facultad de Química, Universidad Nacional Autónoma de México, Mexico City 04510, Mexico; rafaelc@unam.mx (R.C.); hercam@unam.mx (A.H.-C.); 4 Central de Microscopia, ENCB-Instituto Politécnico Nacional, Mexico City 11340, Mexico; eolopez@ipn.mx; 5 Facultad de Ciencias Químicas, Universidad Autónoma Benito Juárez de Oaxaca, Oaxaca 68120, Mexico; arimor10@hotmail.com

**Keywords:** leishmanicidal activity, benzimidazole derivative, ultrastructural studies, ROS production, in vivo activity

## Abstract

Chemotherapy currently available for leishmaniasis treatment has many adverse side effects and drug resistance. Therefore, the identification of new targets and the development of new drugs are urgently needed. Previously, we reported the synthesis of a *N*-(2-methoxyphenyl)-1-methyl-1*H*-benzimidazol-2-amine, named compound **8**, with an IC_50_ value in the micromolar range against *L. mexicana*, it also inhibited 68.27% the activity of recombinant *L. mexicana* arginase. Herein, we report studies carried out to characterize the mechanism of action of compound **8**, as well as its in vivo leishmanicidal activity. It was shown in our ultrastructural studies that compound **8** induces several changes, such as membrane blebbing, the presence of autophagosomes, membrane detachment and mitochondrial and kinetoplast disorganization, among others. Compound **8** triggers the production of ROS and parasite apoptosis. It reduced 71% of the parasite load of *L. mexicana* in an experimental model of cutaneous leishmaniasis in comparison with a control. Altogether, the data obtained suggest the potential use of compound **8** in the treatment of cutaneous leishmaniasis.

## 1. Introduction

Leishmaniasis is a disease caused by protozoan species of the genus *Leishmania* with a wide range of hosts, including man; it is transmitted by the bite of infected female phlebotomine sandflies. It is considered by the World Health Organization (WHO) a neglected tropical disease that affects 98 countries, mainly developing countries in the four continents. The WHO estimated almost 14 million cases of human leishmaniasis worldwide and 700,000 to 1 million new cases annually [1]. There are three main types of leishmaniasis: cutaneous (CL), the most common form of the disease, caused by *L. mexicana* complex in the New World and *L. tropica* and *L. major* in the Old World; mucocutaneous (MCL), the most destructive form of the disease, caused by *L. brazilienzis* complex in countries of the New World; and visceral leishmaniasis (VL), the most severe form of the disease, caused by *L. donovani* in Old World countries and *L. infantum* in Old and New World countries [1]. In the American continent, an average of 55,000 cases of CL and MCL and 3500 cases of VL are recorded each year, with an average mortality rate of 7%. CL is endemic in 18 countries extending from Mexico to Argentina. Recent studies revealed that leishmaniasis produces a disease burden of 2.35 million DALYs (years of life lost due to disability), of which 2.3% are in the Americas [2]. In Mexico, an increase of 60.5% in CL and MCL cases was registered from 2020 to 2021 [3].

The control of leishmaniasis mainly relies on the main drugs available for its treatment, pentavalent antimonials (SbV), Sodium Stiboglucanate (Pentostam) and Meglumine Antimoniate (Glucantime), Amphotericin B (Fungizone) and Miltefosine [4]. Antimonials remain the primary drugs against different forms of leishmaniasis in several regions. The route of administration with the exception of Miltefosine is parenteral, and recently, with the aim of reducing adverse effects, the intralesional administration of SbV has been proposed [5]. However, chemotherapy with these leishmanicidal drugs is not satisfactory since it has many adverse side effects and long-time administrations, as well as high costs and the emergence of parasite drug resistance [6]. These limitations highlight the urgent need for the development of alternative strategies of treatments, new drugs and new parasite targets. 

In this regard, several strategies have been developed as an alternative for leishmaniasis treatment. Liposomes, as nanocarriers for drug delivery, have been used to improve the pharmacokinetic and pharmacodynamic characteristics of leishmanicidal drugs. Indeed, liposomes are well-investigated nanocarriers for drug delivery in macrophage-targeted therapy. For this, liposomes have been surface-modified with Hyaluronic acid (L-HA) or surface-modified with HA-Thiocholesterol (L-HAChol) to encapsulate the quinoxaline derivative, LSPN331, and tested for their activity against *L. amazonensis* amastigotes. The half-maximal inhibitory concentration (IC_50_) values, which is a measure of a drug’s activity, showed that L-HA had lower activity than L-HAChol. Importantly, mice infected with *L. amazonensis* showed that both liposomal formulations were able to target the lesion site after intravenous administration. However, their antileishmanial in vivo activity was not evaluated [7]. The use of nanotechnology has also been investigated. Nanoassemblies made from antimonial drugs through the reaction of the non-ionic surfactant *N*-octanoyl-*N*-methylglucamide with KSb(OH)_6_, named SbL8, were reported. SbL8 forms micelle-like nanostructures in aqueous solution. The administration of SbL8 in mice via the oral route showed greater levels of Sb in the serum and liver when compared with Glucantime^®^ at the same dose. Its antileishmanial activity was demonstrated in murine models of cutaneous leishmaniasis [8]. In addition, drug combination has been used to obtain a synergistic effect to allow the administration of antileishmanial drugs below their original dose limits, treatments with higher tolerance, fewer side effects and shorter treatments; importantly, it has also been considered to achieve a reduction in drug resistance. Associating nanotechnology and combination therapy allowed for the incorporation of Miltefosine into SbL8 nanoassemblies (SbL8/Miltefosine), and its efficacy via the oral route in murine cutaneous leishmaniasis caused by *L. amazonensis* was evaluated. SbL8/Miltefosine, SbL8 Miltefosine or saline were administrated for 30 days. SbL8/Miltefosine nanoassemblies induced a significant reduction in the lesion size growth in comparison with the control (saline) and Miltefosine; however, only SbL8/Miltefosine nanoassemblies significantly decreased the parasite burden in the lesions [9]. On the other hand, the search for new drugs and therapeutic targets has continued. In this regard, the leishmanicidal activity of a preparation of combined alkaloidal extract of *Solanum lycocarpum* (solamargine and solasonine) was demonstrated. Both alkaloids had higher activity against intracellular and extracellular *L. mexicana* parasites than the reference drug sodium stibogluconate. In vivo treatment of C57BL/6 mice with a standardized topical preparation containing both alkaloids induced significant reductions in ear lesion sizes, and parasite counts were calculated at the end of the study using a limiting dilution assay [10]. In addition, the antileishmanial activities of 6,7-dichloro-2,3-diphenylquinoxaline (LSPN329) and 2,3-di-(4-methoxyphenyl)-quinoxaline (LSPN331) against *L. amazonensis* intracellular amastigotes were assessed, and their cytotoxicities were determined. LSPN329 and LSPN331 showed IC_50_ in the range of 16.3 to 19.3 µM, respectively, against *L. amazonensis* intracellular amastigotes in comparison with Miltefosine (2.4 µM). The selectivity index (SI = CC_50_/IC_50_) of LSPN329, LSPN331 and Miltefosine was 12.5, 30.6 and 16.9, respectively. It was suggested that their mechanism of action is related to the mitochondrial changes and the increase in lipid inclusions. However, in vivo studies revealed a lower efficiency of the quinoxaline derivatives to reduce skin lesions after intralesional administration compared with Miltefosine [11]. The inhibition of *L. amazonensis* growth by the calpain inhibitor MDL 28170 was also studied. After 48 h of treatment, the inhibitor exhibited dose-dependent antileishmanial activity, with a 50% lethal dose (LD_50_) of 23.3 µM. MDL 28170 induced short and round parasites. Calpain-like molecules were identified on the cell surface of the parasite [12]. MDL28170 treatment with IC_50_ and two times the IC_50_ doses induced the loss of parasite viability, determined with a resazurin assay, and also triggered the depolarization of the mitochondrial membrane. Scanning and transmission electron microscopic studies revealed alterations in the parasite morphology, some of them resembling apoptotic-like death, including cell shrinking, surface membrane blebs and altered chromatin condensation patterns [13].

With the aim to develop new therapeutic agents, a chloroquinolin derivative, 7-chloro-*N,N*-dimethylquinolin-4-amine, named GF1059, was in vitro tested against *L. infantum* and *L. amazonensis* parasites, which was highly effective against the promastigote and amastigote and with a selectivity index higher than Amphotericin B. Mice treated with 5 mg/kg body weight of GF1059 (50 μL) for 10 days presented a lower parasite load than the control group but higher than Amphotericin B [14]. Novel leishmaniasis therapeutic targets such as the Lmj_04_BRCT domain have been identified. It is linked with *L. major* infectivity and treatment resistance. The structure of this domain was explored using homology modeling and the virtual screening of a set of 266,435 commercially available compounds. Seven compounds selected from the top-ranked hits from the virtual screening were tested in vitro. One compound, CPE2, was the most active against *L. major* promastigotes with an IC_50_ value of 58.13 µM. CPE2 also showed leishmanicidal activity against *L. amazonensis* and *L. infantum* promastigotes (IC_50_ = 62.44 and 101.85 µM, respectively). It also induced a significant decrease in the intracellular parasite load. CPE2 showed no macrophage cytotoxicity at 200 µM. However, its antileishmanial activity was not evaluated [15]. In addition, eight new 4-phenyl-1,3-thiazol-2-amines were synthesized and assayed against *L. amazonensis* promastigotes, and their cytotoxicity was tested against Vero cells. Four out of the eight compounds had IC_50_ values of 20.78 to 53.12 μM and a selectivity index from 4.80 to 26.11. It was proposed that S-methyl-5-thioadenosine phosphorylase is a potential target for these compounds; this enzyme participates in the production of nucleotides and is essential to cell growth and proliferation. These 4-phenyl-1,3-thiazol-2-amines were considered to be good scaffolds for the development of new antileishmanial agents [16]. The polyamine biosynthetic pathway represents an important target since it is essential for parasite survival, and it shows a significant difference from that of mammalian hosts. In this metabolic pathway, *L. mexicana* arginase (LmARG) participates in hydrolyzing L-arginine to produce L-ornithine, the main source for polyamine synthesis and it is considered a therapeutic target for the treatment of leishmaniasis since it shares only 39–43% identity with human ARG [17,18]. Previously, our group demonstrated the leishmanicidal activity of a new *N*-(2-methoxyphenyl)-1-methyl-1*H*-benzimidazol-2-amine, named compound **8**, with IC_50_ values in the micromolar range mainly against the promastigote and amastigote of *L. mexicana* (3.21 and 0.26 µM, respectively) and a higher selectivity index in comparison with Miltefosine and Amphotericin B. It also inhibited 68.27% of the activity of recombinant LmARG [19]. In the present study, we aimed to continue the characterization of the leishmanicidal activity of compound **8**. To achieve this, an ultrastructural analysis of compound **8**-treated parasites was performed. The production of reactive oxygen species and the induction of an apoptotic process were also analyzed. In addition, the in vivo activity of compound **8** was evaluated in an experimental model of CL.

## 2. Results

### 2.1. Scanning and Transmission Electron Microscopy 

**Figure 1:**
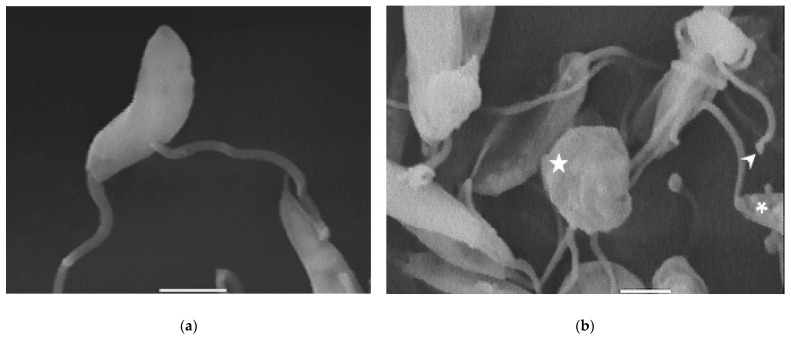

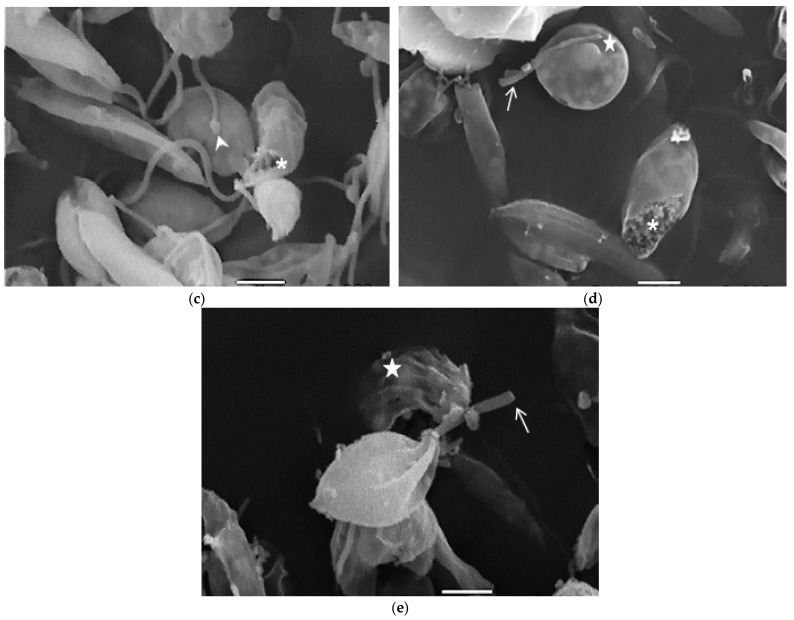
SEM analysis of *L. mexicana* promastigotes: non-treated (**a**) or treated with compound **8** at 3.2 µM for 6 h (**b**,**c**) and 12 h (**d**,**e**). Control promastigotes show the typical morphology (**a**). Treated parasites show distorted shapes, surface damage (**b**–**d**) (asterisk), modification to the flagella tip (arrowhead) (**b**,**c**), shortened flagella (arrow) (**d**,**e**) and membrane blebbing (star) (**b**,**d**,**e**). (bar = 2 µm). magnification × 8000.

Compound **8** induced several ultrastructural changes in *L. mexicana* promastigotes, as evidenced by SEM (Figure 1). SEM analysis of the non-treated parasites showed a typical elongate morphology of promastigotes with their anterior flagella (Figure 1a); meanwhile, the treated parasites showed a rounded shape (Figure 1b–e), profound alterations in their membranes (Figure 1b–d), membrane blebbing (Figure 1b,d,e) and flagella length reduction as well as a bulbous structure at the flagella tip (Figure 1b–e).

### 2.2. Transmission Electronic Microscopy

**Figure 2:**
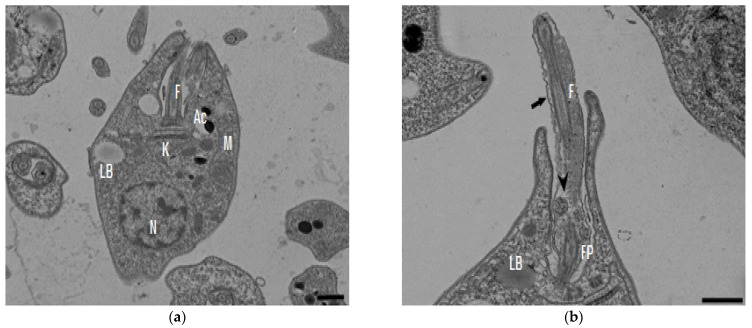

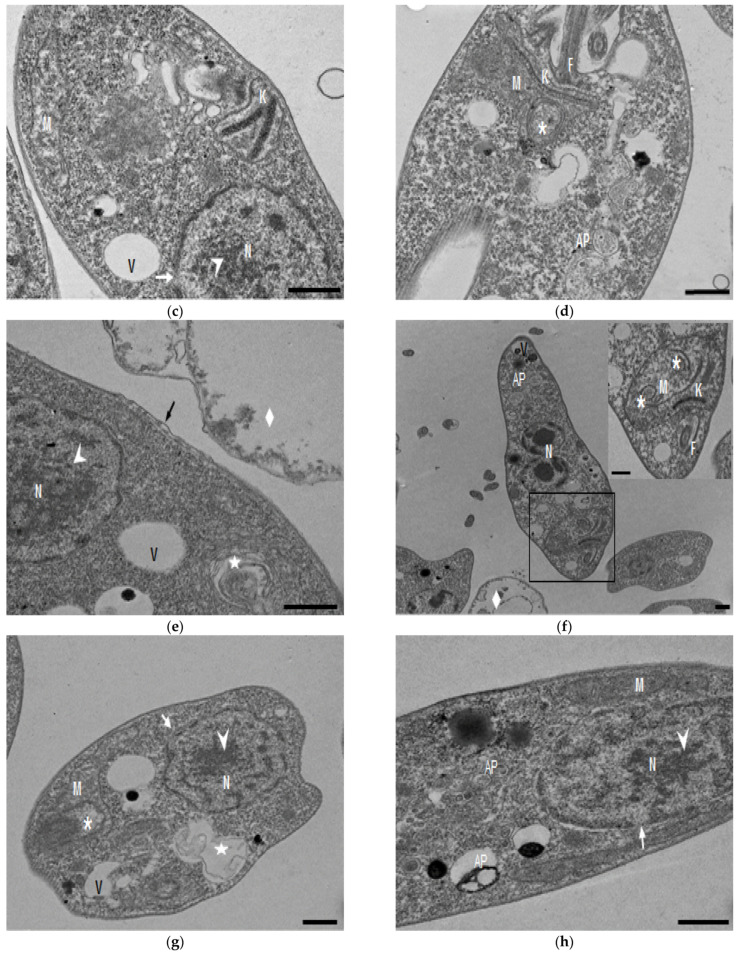
TEM analysis of *L. mexicana* promastigotes: non-treated (**a**) and treated with compound **8** at 0.32 µM 6 h (**b**–**d**) and 3.2 µM for 12 h (**e**–**h**). Control parasites show a normal morphology. Parasites treated with compound **8** show the presence of autophagosomes (APs) (**d**,**f**,**h**) and several alterations such as flagellar membrane detachment (black arrow) and exocytic activity in the flagellar pocket (black arrowhead) (**b**), kinetoplast structural alterations (**c**), mitochondrion swelling and concentric membranes inside (asterisks) (**d**,**f**,**g**), chromatin condensation (white arrowhead) (**c**,**e**,**g**,**h**), rupture of nuclear membrane (white arrow) (**c**,**g**,**h**), plasmatic membrane detachment (black arrow) (**e**) and cytoplasmic myelin figures (white stars) (**e**,**g**); loss of cell integrity and cytosolic content (white rhombus) (**e**,**f**); nucleus (N), mitochondrion (M), flagella (F), flagellar pocket (FP), kinetoplast (K), acidocalcisome (Ac), lipid body (LB), vacuole (V) (bar = 0.5 µm).

In the TEM studies (Figure 2), the control parasites were found to have plasma membranes and their characteristic structures without any damage (Figure 2a). However, the parasites treated for 6 h at 0.32 µM showed some early effects induced by compound **8** (Figure 2b–d). Some ultrastructural changes were evident such as the formation of autophagosomes and mitochondrion swelling with the presence of concentric membranal structures inside as well as an exocytic activity in the flagellar pocket, detachment of the flagellar membrane and alterations in the kinetoplast. Meanwhile, at 3.2 µM for 12 h (Figure 2e–h), the treated parasites showed even more profound changes such as the presence of increased vacuolization and autophagosomes, cytoplasmic myelin-like figures, plasma membrane detachment, rupture of the nuclear membrane and abnormal chromatin condensation. Total mitochondrion disorganization with swelling and concentrical membranes inside and, in some cases, the loss of cell integrity and cytosolic content were also observed. 

### 2.3. Measurement of Reactive Oxygen Species (ROS)

**Figure 3:**
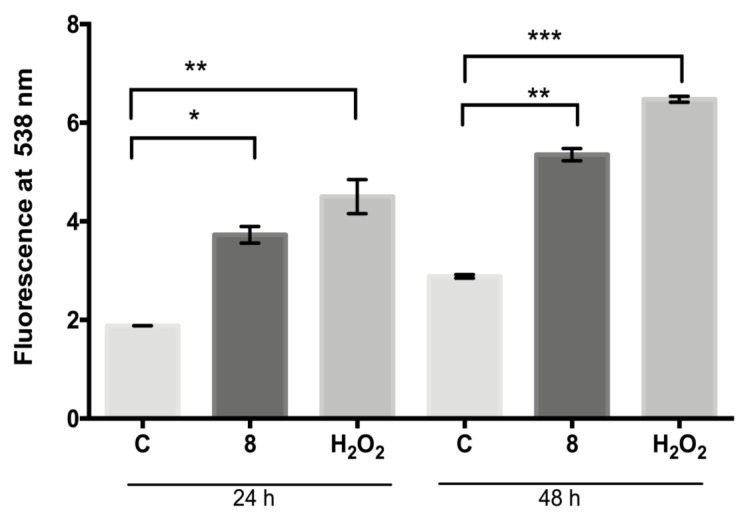
Generation of ROS by *L. mexicana* promastigotes: control or treated with compound **8** (IC_50_ value) and hydrogen peroxide (100 µM) for 24 and 48 h. Values are expressed as units of fluorescence emitted by the probe H_2_DFFDA at 538 nm. * *p* ≤ 0.004. ** *p* ≤ 0.001. *** *p* ≤ 0.0002.

The fluorescent dye H_2_DFFDA was used to measure the intracellular generation of ROS induced by compound **8** at 24 h and 48 h (Figure 3). The ROS levels (fluorescent units at 538 nm) determined in the control parasites were 1.8 at 24 h and 2.9 at 48 h, respectively; meanwhile, the ROS levels in the compound **8**-treated parasites increased to 3.7 and 5.3 at 24 and 48 h, respectively. The ROS levels increase induced with compound **8** at both time intervals analyzed was significantly different in comparison with the control (*p* ≤ 0.004 and *p* ≤ 0.001, respectively). Hydrogen peroxide (positive control) significantly increased the ROS production to 4.5 and 5.3 after treatment for 24 and 48 h, respectively, with respect to the control (*p* ≤ 0.001 and *p* ≤ 0.0002, respectively). 

### 2.4. Flow Cytometry Analysis

**Figure 4:**
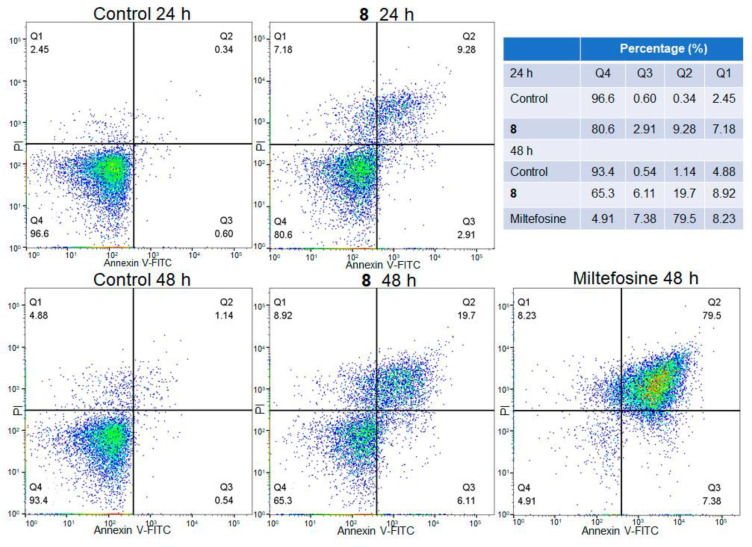
Flow cytometry analysis of *L. mexicana* promastigotes: control and treated with compound **8** (IC_50_) and Miltefosine at different times. Q4 viable cells (FITC−/PI−), Q3 early apoptotic cells (FITC+/PI−), Q2 late apoptotic cell (FITC+/PI+) and Q1 necrotic cells (FITC−/PI+). Cell density is color-coded, with red and green denoting high density and blue low density.

The biparametric representation (Annexin V-FITC versus PI) obtained from the flow cytometry assay of *L. mexicana* promastigotes treated with compound **8** (IC_50_ value) at different times (24 and 48 h) is shown in Figure 4. Parasites without treatment and parasites treated with Miltefosine were included as negative and positive controls, respectively. The control parasites showed 96.6% and 93.4% viability at 24 and 48 h; meanwhile, the promastigotes treated with compound **8** showed lower numbers of viable cells and a higher number of apoptotic cells in comparison with the control parasites. Parasite viability from 24 h to 48 h of treatment was reduced from 80.6% to 65.3%; meanwhile, early apoptotic cells increased from 2.91% to 6.11%, and late apoptotic cells increased from 9.28% to 19.7%. The positive control showed a high percentage of parasites (79.5%) in the late apoptotic phase.

### 2.5. In Vivo Leishmanicidal Activity of Compound ***8***

**Figure 5:**
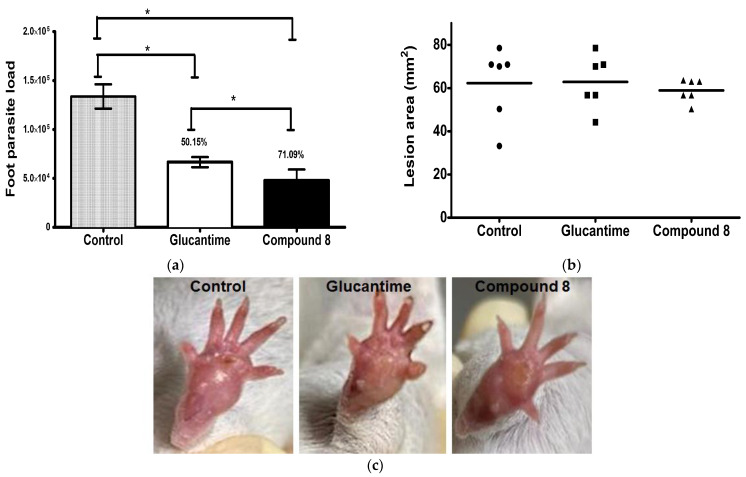
Footpad parasite load and lesion area in *L. mexicana*-infected mice treated with compound **8** and Glucantime. BALB/c mice were subcutaneously infected with 1 × 10^6^ stationary promastigotes of *L. mexicana*. Compound **8** and Glucantime at 30 mg/kg were administered on day 49 pi via the intraperitoneal route on alternate days to complete seven administrations. (**a**) Footpads were aseptically removed one week after the end of treatment and parasite load was determined by limiting dilution * *p* ≤ 0.001. (**b**) Lesion areas were measured before the animals were sacrificed and determined as described in the methodology. (**c**) Macroscopic images of the footpad of infected animals: control mice and mice treated with Glucantime and Compound **8**.

The leishmanicidal activity of compound **8** was evaluated in an experimental model of CL. It was shown that compound **8** and Glucantime have an important activity against *L. mexicana* in comparison with non-treated infected mice. Compound **8** significantly reduced the parasite load by 71% in comparison with Glucantime (50%) and the control (*p* ≤ 0.001) (Figure 5a). There were not dead animals. The lesion area (mm^2^) of the animals treated with compound **8** and Glucantime determined one week after the end of the treatment showed no difference in comparison with the control group (Figure 5b). The lesion area of the non-treated infected mice and the infected mice treated with compound **8** and Glucantime was measured on day 49 post-infection; when the drug administration was initiated to day 63, the footpads were removed, as shown in Supplementary Material (Figure S1). Macroscopic images of the footpad of the infected animals are provided for the control mice and the infected mice treated with Glucantime and compound **8** (Figure 5c). 

## 3. Discussion

In the present study, with an interest in continuing the characterization of compound **8**’s leishmanicidal activity, SEM studies were performed. It was demonstrated that compound **8** induced important ultrastructural changes in *L. mexicana* promastigotes such as membrane blebbing resembling an apoptotic-like process [20] and alterations in the flagella structure. In addition, TEM studies revealed alterations in the kinetoplast structure and profound mitochondrion damage including swelling and the presence of concentric membranes inside the organelle. We also observed cytoplasmic myelin-like figures indicative of an autophagy process, among other changes. Similar findings were described when *L. amazonensis* promastigotes were treated with Miltefosine [20], antifungal azoles [21] and calpains inhibitors [22]. In trypanosomastids, severe mitochondrion damage as well as an intense autophagy process and subsequent apoptosis cell death was related to a significant increase in reactive oxygen and nitrogen species (ROS and RNS) [23]. Parasites are exposed to extracellular ROS during their entire life cycle in the insect vector, as well as during the invasion of mammalian host cells [24], but ROS are also produced within the parasite through its electron transport chain or drug detoxification. In the present study, it was demonstrated that compound **8** triggers the production of ROS and parasite apoptosis, indicating that intracellular ROS production induced with compound **8** could be related to mitochondrial damage, as well as membrane blebbing and myelin-like figures observed in the SEM and TEM studies. Parasite apoptosis induced with compound **8** was demonstrated in the flow cytometry studies. 

In a previous study, the inhibitory activity of compound **8** on LmARG was demonstrated using both molecular docking studies and experimental assays [19]. LmARG is involved in polyamine synthesis converting L-arginine to L-ornithine and, therefore, in the trypanothione (TSH2) pathway and the parasite detoxification [18]. It is well-known that an increase in parasite thiol production results in a greater ability to deal with oxidative stress. In this regard, the inhibition of LmARG could alter redox balance and parasite antioxidant levels. Data obtained in this study showed that compound **8** induced an increase in parasite ROS production, indicating that the redox metabolism of parasites was compromised, which may be related to the ultrastructural damage observed. In fact, parasite oxidative metabolism has been proposed as an excellent target to develop new drugs [25].

In addition, the in vivo activity of compound **8** against *L. mexicana* was demonstrated in an experimental model of CL. It showed higher leishmanicidal activity than the reference drug, Glucantime, suggesting its potential use as leishmanicidal. 

In general terms, compound **8** showed higher activity against *L. mexicana* promastigotes and amastigotes (3.21 and 0.26 µM, respectively) than Miltefosine (15.34 and 0.51 µM, respectively) and, although it was not more active than Miltefosine against the promastigote and amastigote of *L. brasiliensis* and *L. donovani*, its selectivity index was higher. LmARG was proposed to be the therapeutic target of compound 8 [19]. The leishmanicidal activity of compound **8** compared with other new therapeutic agents such as CPE2 is higher. CPE2 was in vitro tested against *L. amazonensis*, which is also causative of cutaneous leishmaniasis, and obtained the highest reduction in macrophage parasite load at 100 µM and its IC_50_ was 62.44 µM against the promastigote. CPE2 recognizes the BRCT domain in *L. major*, *L. amazonensis* and *L. infantum*, which is linked with infectivity and treatment resistance [15]. In the case of new 4-phenyl-1,3-thiazol-2-amines, IC_50_ values against the *L. amazonensis* promastigote were in the range of 20.78 to 53.12 μM, and the selectivity index was from 4.80 to 26.11. S-methyl-5-thioadenosine phosphorylase was proposed as a potential target for these compounds, which plays an important role in the production of nucleotides [16]. Other tested compounds such as LSPN329 and LSPN331 against *L. amazonensis* intracellular amastigotes showed IC_50_ values of 16.3 and 19.3 µM. The leishmanicidal activity of LSPN329 and LSPN331 was evaluated in a BALB/c model of mucocutaneous leishmaniasis. Compounds at 5 mg/kg body weight administered intralesionally daily for 3 weeks induced a significant decrease in lesion thickness in infected footpads compared with the control; however, the parasite load in the lesion was not evaluated [11]. Herein, the leishmanicidal activity of compound **8** was evaluated in BALB/c mice infected with *L. mexicana* at 30 mg/Kg body weight administered intraperitoneally. The dose was administered on alternate days to complete seven administrations in comparison with some other studies where the drug was evaluated at lower concentrations, but its administration was daily for 3 weeks [11] or even daily for 30 days [9]. The parasite load reduction induced with compound **8** was calculated using a limiting dilution assay. Haghdoust et al. [26] found significant correlations between the parasite burden determination with assays using direct fluorescent microscopy of *L. major* expressing the enhanced green fluorescent protein and either limiting dilution assay (r = 0.976, *p* < 0.0001) or quantitative real-time PCR assay (r = 0.857, *p* < 0.001). The parasite load reduction obtained with compound **8** (71.09%) was higher than the control and significantly different than Glucantime (50.15%), and no dead animals were registered during the treatment. Regarding the lesion size, no significant difference was obtained when Glucantime or compound **8** was administered. For BALB/c mice infected with *L. amazonensis* in footpads and treated with Glucantime at 30 mg/kg/intralesional route/every four days four times followed by 10 weeks, it was reported that the lesion size did not reduce [27]. To confirm the potential use of compound **8** in the treatment of leishmaniasis, further pharmacokinetic and toxicological studies, among others, are required. 

The importance of counting on a new drug is highlighted by the emergence of parasite resistance to all known anti-*Leishmania* drugs [6,28,29]. Compound **8** was previously shown to have optimal requirements for druggability [19]. 

## 4. Materials and Methods

### 4.1. Chemical Synthesis

**Scheme 1:**
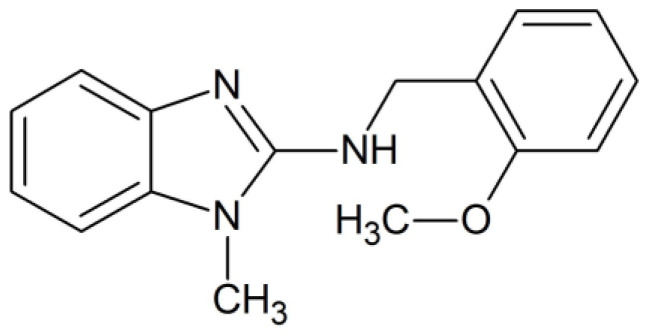
Chemical structure of *N*-(2-methoxyphenyl)-1-methyl-1*H*-benzimidazol-2-amine, named compound **8**.

The synthesis of the *N*-(2-methoxyphenyl)-1-methyl-1*H*-benzimidazol-2-amine, compound **8**, was prepared as previously described by our group [19]. The chemical structure of compound **8** is presented in Scheme 1. 

### 4.2. Parasites

Promastigotes of *L. mexicana* (strain MNYC/BZ/62/M379) were grown in Schneider’s Insect Medium (Sigma-Aldrich) supplemented with 10% heat-inactivated Fetal Bovine Serum (FBS) and 100 U/mL of penicillin plus 100 μg/mL of streptomycin (Sigma-Aldrich Co., St. Louis, MO, USA), in 25 mL culture flasks.

### 4.3. Scanning Electron Microscopy (SEM)

Parasite promastigotes (1 × 10^8^) were incubated with compound **8** at the IC_50_ value for 6 and 12 h at 26 °C. Non-treated parasites were included as a negative control. The control and treated promastigotes were fixed for 1 h in a solution containing 2.5% glutaraldehyde in 0.1 M cacodylate buffer (pH 7.2) and post-fixed for 1 h with 1% osmium tetroxide (OsO_4_) in 0.1 M cacodylate buffer and then dehydrated with ethanol. The samples were subjected to critical point drying in a CO_2_ atmosphere in a Samdry-780A apparatus (Tousimis Research, Rockville, MD, USA) and gold coated in a Denton Vacuum Desk II (INXS, Inc., Delray Beach, FL, USA). Coverslips containing the samples were attached to aluminum holders and analyzed using a scanning electron microscope model JSM5800LV (JEOL, Tokyo, Japan).

### 4.4. Transmission Electron Microscopy (TEM)

*L. mexicana* promastigotes (1 × 10^8^) were treated with compound **8** at 0.32 µM (1/10 IC_50_ value) for 6 h and 3.2 µM (IC_50_ value) for 12 h at 26 °C. Non-treated promastigotes were included as a control. The parasites were fixed in 2% glutaraldehyde and 4% paraformaldehyde in PHEM buffer (5 mM MgCl_2_, 70 mM KCl, 20 Mm Hepes, 60 mM Pipes and 10 mM EGTA) pH 7.2 for 1 h. The parasites were washed twice in PBS pH 7.2 and post-fixed in 1% osmium tetroxide supplemented with 0.8% potassium ferricyanide. After this, the parasites were dehydrated in an ascending series of ethanol and embedded in EMbed 812 resin (Electron Microscopy Sciences, Hatfield, PA, USA). Ultrathin sections were stained with 5% uranyl acetate and Reynold’s solution and observed under a JEOL JEM-1010 transmission electron microscope.

### 4.5. Measurement of Reactive Oxygen Species (ROS)

The oxidative fluorescent dye H_2_DFFDA (Invitrogen, Eugene, OR, USA) was used to evaluate the ROS production induced with compound **8**. *Leishmania mexicana* promastigotes were treated with compound **8** (IC_50_ value) for 24 h and 48 h at 26 °C and centrifuged at 327× *g* for 10 min. Then, 1 × 10^7^ parasites/mL were incubated with 200 µL of H_2_DFFDA (10 µM) for 60 min in the dark at room temperature. Hydrogen peroxide (100 µM) and non-treated parasites were used as positive and negative controls, respectively. The H_2_DFFDA-fluorescence intensity was measured at 485 nm excitation and 538 nm emission wavelength with a cytofluorimeter (Fluoroskan Ascent FL, Thermo Labsystems, Waltham, MA, USA). 

### 4.6. Annexin V-FITC Assay

Parasites were grown in the presence of compound **8** (at the IC_50_ value) for 24 and 48 h at 26 °C. Miltefosine (15 µM for 48 h) was included as an apoptotic reference drug. Phosphatidylserine externalization was analyzed using Annexin V-FITC Kit (Beckman Coulter Company, Marseille, France), following the manufacturer’s instructions. Plasma membrane integrity was determined by the DNA-intercalating capabilities of propidium iodide (PI). The parasite samples were analyzed with flow cytometry (MACSQuant Analyzer, Miltenyi Biotec, Auburn, CA, USA). The analysis was performed with Flowjo version 10 Software.

### 4.7. Evaluation of the In Vivo Leishmanicidal Activity of Compound ***8***

Groups of six male BALB/c mice, 4 weeks old, were randomly sorted. The animals were allocated in plastic cages in a 12 h dark–light cycle with controlled temperature (25 °C) and humidity (70%). Water and food were unrestricted throughout this study. The animals that were previously anesthetized with sodium pentobarbital and 1 × 10^6^ stationary promastigotes of *L. mexicana* in Schneider’s Insect Medium were injected subcutaneously in the left hind footpad. The right hind footpad was used as a negative control. Forty-nine days post-infection (pi), groups were organized as follows: group 1 was treated with compound **8** (resuspended in DMSO solution as vehicle); group 2 was treated with the reference drug, Glucantime (Glucantime^®^; Sanofi-Aventis, São Paulo, Brazil); and group 3 was the infected control injected with vehicle solution. The drugs were administered at 30 mg/kg body weight via the intraperitoneal route on alternate days to complete seven administrations. One week after the end of treatment (day 63 pi), the animals were sacrificed to assess parasite load in the infected footpad and to measure the lesion size. Lesion area (in mm^2^) was determined by measuring the two crossed diameters of the footpad with a digital caliper and calculated with the formula previously recommended by Muñoz-Durango et al., 2022 [30]. The footpads were aseptically removed and macerated in Schneider’s Insect Medium, and the parasite load was determined with limiting dilution assays, as previously described [31,32]. Briefly, homogenates were serially diluted tenfold in 96-well flat-bottomed culture plates and then incubated at 26 °C. The parasite load was determined by the highest dilution at which promastigotes grew after 10 days of incubation. Data are expressed as average ± SD.

### 4.8. Statistical Analysis 

Statistical analysis was performed using Student’s *t*-test with GraphPad Prism software version 5.01 (San Diego, CA, USA). Values expressed as mean ± SD were considered to be statistically significant with *p* ≤ 0.05 values.

## 5. Conclusions

The data obtained in the present study indicate that the leishmanicidal activity of compound **8** induces damage to several parasite structures such as the mitochondria. In addition, it triggers parasite intracellular ROS production and apoptosis. The effects induced with compound **8** may account without a doubt to its leishmanicidal activity demonstrated in an experimental model of CL. Further pharmacokinetic and toxicological studies, among others, are required to confirm the potential use of compound **8** in the treatment of leishmaniasis. 

## Data Availability

No new data were created or analyzed in this study. Data sharing is not applicable to this article.

## Supplementary Materials

The following supporting information can be downloaded at: https://www.mdpi.com/article/10.3390/ijms25010659/s1.
