# Supplementary material for: Characterization of the Effect of N-(2-Methoxyphenyl)-1-methyl-1H-benzimidazol-2-amine, Compound 8, against Leishmania mexicana and Its In Vivo Leishmanicidal Activity
Source: Int J Mol Sci. 2024 Jan 4;25(1):659. doi: 10.3390/ijms25010659 (PMC10779428; doi:10.3390/ijms25010659)
Supplement: Supplementary file 1 [file ijms-25-00659-s001.zip › ijms-2776780-supplementary.pdf]

International Journal of *Molecular Sciences*

MDPI

Supplementary Material

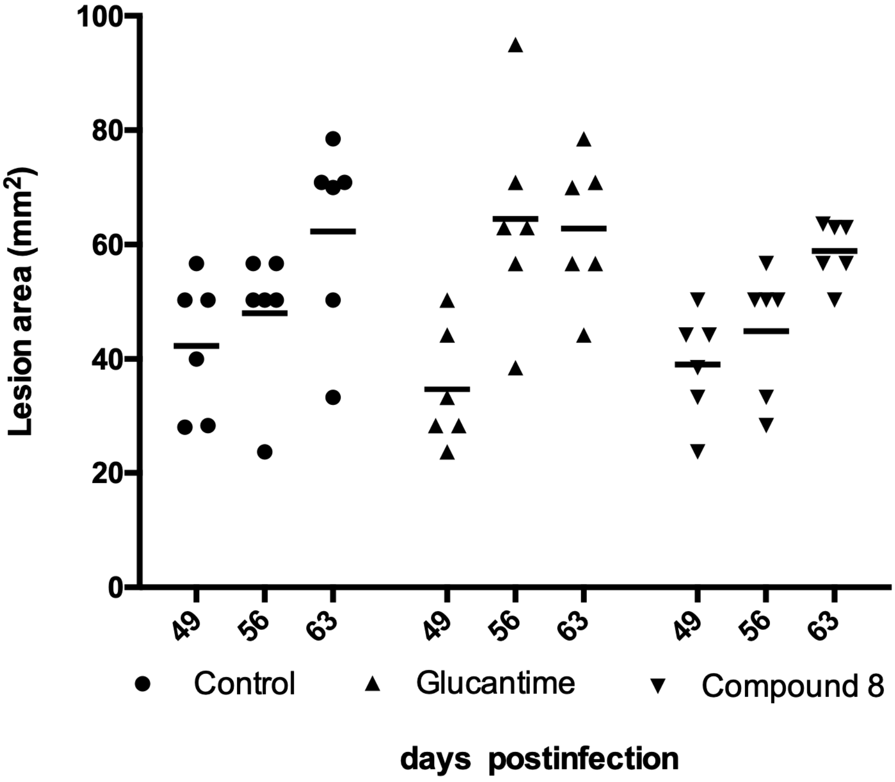


**Figure S1.** Lesion size localized in the footpads of non-treated and treated mice infected with *L. mexicana.* The lesion area of non-treated infected mice (•) and infected mice treated with compound **8** (▾) and Glucantime (▴) were measured at day 49 post infection, when the drugs administration was initiated to day 63 when footpads were removed.
